# VSS-Hi-C: variance-stabilized signals for chromatin contacts

**DOI:** 10.1093/bioinformatics/btae715

**Published:** 2024-12-10

**Authors:** Neda Shokraneh Kenari, Faezeh Bayat, Maxwell W Libbrecht

**Affiliations:** Department of Computing Science, Simon Fraser University, Burnaby, BC, V5A 1S6, Canada; Department of Computing Science, Simon Fraser University, Burnaby, BC, V5A 1S6, Canada; Department of Computing Science, Simon Fraser University, Burnaby, BC, V5A 1S6, Canada

## Abstract

**Motivation:**

The genome-wide chromosome conformation capture assay Hi-C is widely used to study chromatin 3D structures and their functional implications. Read counts from Hi-C indicate the strength of chromatin contact between each pair of genomic loci. These read counts are heteroskedastic: that is, a difference between the interaction frequency of 0 and 100 is much more significant than a difference between the interaction frequency of 1000 and 1100. This property impedes visualization and downstream analysis because it violates the Gaussian variable assumption of many computational tools. Thus heuristic transformations aimed at stabilizing the variance of signals like the shifted-log transformation are typically applied to data before its visualization and inputting to models with Gaussian assumption. However, such heuristic transformations cannot fully stabilize the variance because of their restrictive assumptions about the mean–variance relationship in the data.

**Results:**

Here, we present VSS-Hi-C, a data-driven variance stabilization method for Hi-C data. We show that VSS-Hi-C signals have a unit variance improving visualization of Hi-C, for example in heatmap contact maps. VSS-Hi-C signals also improve the performance of subcompartment callers relying on Gaussian observations. VSS-Hi-C is implemented as an R package and can be used for variance stabilization of different genomic and epigenomic data types with two replicates available.

**Availability and implementation:**

https://github.com/nedashokraneh/vssHiC.

## 1 Introduction

The 3D organization of chromatin in the nucleus plays a key role in many cellular processes. Chromatin conformation can control transcription through mechanisms including positioning genes within nuclear components—such as nuclear speckles—enriched with transcription factors (Chen *et al.* 2018), organizing genomic elements into topological association domains (TADs) (Stadhouders *et al.* 2019), colocalizing genes and their enhancers, and facilitating repression (Szabo *et al.* 2019). The genome-wide chromosome conformation capture assay Hi-C assesses chromatin conformation by measuring the frequency of contacts or interactions between pairs of genomic bins. Inferring biological insights from Hi-C data requires accurate computational analysis methods.

Researchers have developed computational tools for processing Hi-C data into a format useful for downstream analysis, including tools for read aggregation (Durand *et al.* 2016, Abdennur and Mirny 2020) and bias normalization (Imakaev *et al.* 2012, Knight and Ruiz 2013). These tools output a matrix of contact counts that measures the contact strength of each pair of genomic positions. However, similar to other sequencing-based measurements, these signals are heteroskedastic; that is, a difference between the interaction frequency of 0 and 100 is much more significant than a difference between the interaction frequency of 1000 and 1100 (Motakis *et al.* 2006, Anders and Huber 2010, Ahlmann-Eltze and Huber 2023).

This property of heteroskedasticity impedes visualization and downstream analyses. For example, a heatmap visualization of raw Hi-C counts has its color map dominated by large outliers, making it impossible to see subtle contact patterns important for loops and compartments. Likewise, many existing analysis methods (Yaffe and Tanay 2011, Rao *et al.* 2014) assume signals follow a Gaussian distribution because doing so is easy to implement, but this assumption is inaccurate for heteroskedastic signals. The same issue also afflicts existing machine learning models that minimize mean-squared error (Zhang *et al.* 2018, Dimmick 2020), as doing so is mathematically equivalent to maximizing the log-likelihood of a Gaussian model. Also, some statistical methods for calling TADs rely on Gaussian data (Lévy-Leduc *et al.* 2014) and variance-stabilizing transformation is necessary before applying these methods.

Two approaches have been proposed to handle heteroskedastic signals. One strategy to address heteroskedasticity is to use a non-Gaussian model, such as a negative binomial distribution, which explicitly accounts for the mean-variance relationship. However, such models can be hard to implement and optimize. Instead, many existing approaches first aim to stabilize variance with a transformation, such as a heuristic shifted log (log(x+1)) and asinh ($\text{ln}(x+\sqrt{x^{2}+1})$). However, such transformations assume a specific mean–variance relationship (e.g. log assumes a quadratic relationship) and thus do not always successfully stabilize variance (Ahlmann-Eltze and Huber 2023).

Thus transformation methods for other data types typically apply a data-driven variance-stabilizing transformation. For example, researchers typically stabilize the variance of RNA-seq using the data-driven variance-stabilizing transformation approach in DESeq (Anders and Huber 2010) (see supplementary Section S1.2 for a review of all previously proposed transformations). Similarly, we recently proposed the delta method-based approach VSS (Bayat and Libbrecht 2021) that stabilizes the variance of 1D genomic signals such as ChIP-seq.

Here, we propose a method, VSS-Hi-C, for stabilizing the variance of Hi-C contact signals. Our contributions presented here are as follows. First, we adapted the VSS method to extend it to handle 2D Hi-C contact matrices. Second, we present a user-friendly R package that provides an interface with an easy-to-use class (InteractionSet) and data format (.cool) for Hi-C data. Third, we propose three evaluation metrics that measure the downstream impact of heteroskedasticity on Hi-C analysis and benchmarked all approaches for Hi-C signals. We found that VSS-Hi-C signals have more stable variance compared to all other transformations and produce informative visualization of chromatin structures at different scales, and we found that VSS-Hi-C signals outperform or perform comparably to other transformations in terms of identifying more biologically validated subcompartments.

## 2 Materials and methods

### 2.1 Problem definition

The read counts from the Hi-C assay represent interaction frequencies between pairs of genomic bins. As mentioned above, these read counts do not have constant variance, meaning we expect an observed interaction between a pair of genomic bins with an observed interaction of 1000 reads to have different (usually higher) uncertainty than another pair of genomic bins with 100 reads. VSS-Hi-C utilizes a Hi-C experiment with two biological replicates to estimate the mean–variance relationship in the experiment and derive a variance-stabilizing transformation.

**Figure 1.**
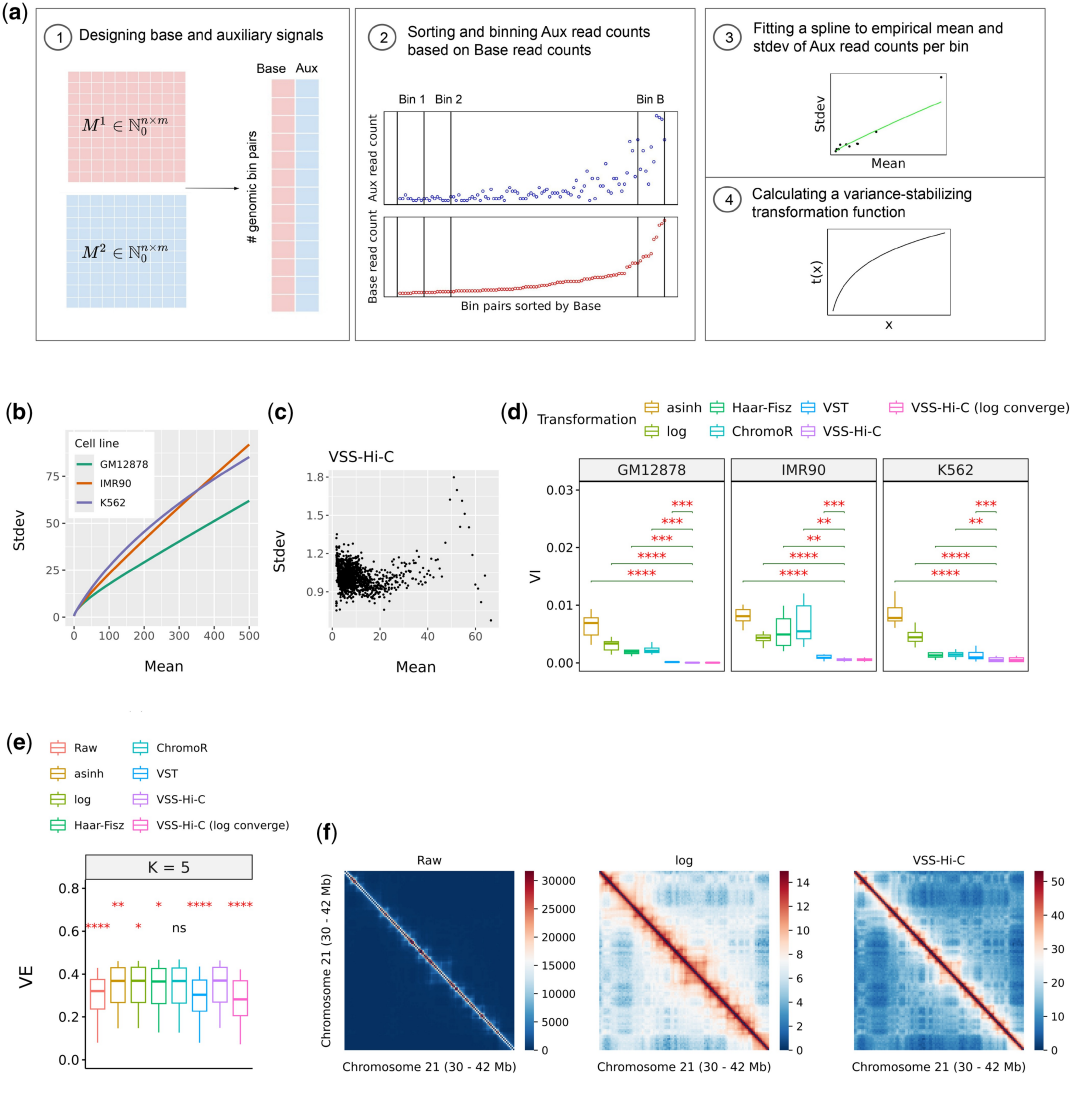
(a) Overview of VSS-Hi-C for variance stabilization of Hi-C data. (b) Learned mean-stdev curves for three Hi-C experiments, each from a different cell line. (c) Standard deviation (stdev) vs mean for VSS-Hi-C signals. This plot is for chromosome 13 of the GM12878 cell line. (d) Variance instability (VI) of transformed signals for three cell lines. Each box represents VIs for chromosomes 13 to 22. (e) Variance explained (VE) of epigenomic features given subcompartment annotations for odd chromosomes by applying HMM on raw and transformed odd-even inter-chromosomal matrix. *K* indicates the number of subcompartment types in the annotation. Each box represents 12 VEs corresponding to 12 epigenomic features for a specific transformation and *K*. This experiment is on the GM12878 cell line. (f) Comparison of heatmap visualization of raw, log-transformed and VSS-Hi-C signals with linear color scale. The heatmap visualizations correspond to the GM12878 cell line. In (d) and (e), Asterisks indicate the significance of paired one-sided *t*-tests with an alternative hypothesis: VSS-Hi-C signals have less VI (greater VE) compared to other transformed signals. *, **, ***, **** represent *P* <0.05, 0.01, 0.001, and 0.0001, respectively.

More formally, the inputs are two Hi-C contact matrices $M^{1},M^{2}\in N^{n\times m}$ corresponding to interaction frequencies between chromosomes *c*1 and *c*2 from two biological replicates (*n* and *m* are the number of genomic bins in chromosomes *c*1 and *c*2, respectively). VSS-Hi-C estimates the mean–variance relationship in data (Fig. 1b), derives a variance-stabilizing transformation *t*(*x*) (Supplementary Fig. S1b) and outputs transformed contact matrices $t(M^{1})$ and $t(M^{2})$ which have a unit variance; that is $\text{var}(M_{i,j})=1$ regardless of the magnitude of $M_{i,j}$ (Fig. 1c).

VSS-Hi-C stems from a general idea for variance-stabilizing transformation, a delta method. First, we briefly explain a delta method and then describe VSS-Hi-C in detail.

### 2.2 Variance-stabilizing transformation based on the Delta method

The delta method is a general method for approximating a probability distribution of a function of an asymptotically normal random variable with a known variance. Let *X* be a random variable with E[X]=μ and $\text{Var}[X]=\sigma^{2}$. A delta method approximates the mean and variance of Y=g(X) where *g* is any function that its first derivative at *μ*, g′(μ), exists and is non-zero. A first-order Taylor approximation for Y=g(X) is:

(1) $$\begin{array}{l} Y=g(X)\approx g(\mu )+g\prime (\mu )(X-\mu )\\ \Rightarrow E[Y]\approx g(\mu ),\text{Var}[Y]\approx \sigma^{2}g\prime {\left(\mu \right)}^{2}. \end{array}$$

Considering there is a known relationship between the mean and variance of a random variable (E[X]=μ and Var[X]=h(μ)), the goal is to find a function *g* such that Y=g(X) has a variance independent of *μ*. Forcing the condition $\text{Var}[Y]\approx h(\mu )g\prime {\left(\mu \right)}^{2}=C$, where *C* is a constant, implies a differential equation:

(2) $$\frac{dg}{d\mu }=\frac{C}{\sqrt{h(\mu )}}\;\Rightarrow \;g(\mu )=\int \frac{C\;d\mu }{\sqrt{h(\mu )}}.$$

Thus we can construct a variance-stabilizing transformation function given any known mean-variance relationship. There are well-known approximate variance-stabilizing transformations for probability distributions with known mean–variance relationships. For example, the negative binomial (gamma-Poisson) distribution commonly used to model RNA-seq and scRNA-seq counts has a quadratic mean–variance relationship $\text{Var}[Y]=\mu +\alpha \mu^{2}$, where *μ* and *α* are the mean and overdispersion of the distribution respectively. Applying a delta method implies an approximate variance-stabilizing transformation $g(y)=\text{log}(y+y_{0})$, which is the widely-used shifted logarithm transform.

To avoid assuming a specific mean–variance relationship, we estimate the mean–variance relationship empirically and use Equation (2) to find a variance-stabilizing transformation *g*, following the previously-described method VSS for 1D genomic data sets (Bayat and Libbrecht 2021).

### 2.3 VSS-Hi-C

VSS-Hi-C has four steps to find a data-driven variance-stabilizing transformation (Fig. 1a):

Step 1: We designate *M*_1_ as the “base” replicate and *M*_2_ as “auxiliary” replicate and design a two-column matrix as follows. For intra-chromosomal matrices (c1=c2), we flatten all the elements in and above the main diagonal of matrices and for inter-chromosomal contact matrices c1≠c2, we flatten whole matrices to fill the base-aux matrix. Each row of the base-aux matrix represents two observations of one pair of genomic bins (Fig. 1a-1).

Step 2: The purpose of constructing a base-aux matrix is to find pairs of genomic bins whose interaction frequencies are likely from the same distribution. For that purpose, we use base observations to order the base-aux matrix, group aux observations into equal-size bins, and assume that pairs of genomic bins within each bin have a similar variance for the purposes of learning (Fig. 1a-2).

More formally, define $X\in N_{0}^{BP\times 2}$ as a base-aux matrix where *BP* is a number of genomic bin pairs. We sort *X* based on its first column (base observations) into $\hat{X}$. Given a bin size of *s*, we group the rows of $\hat{X}$ into $B=\left\lceil \frac{BP}{s}\right\rceil$ bins.

In VSS-Hi-C, we only use two replicates and choose one replicate as a base and another as an auxiliary signal. This can be changed to support more than two replicates similar to VSS, however, we have not found any Hi-C experiment with more than two replicates.

Step 3: Assuming that aux read counts within one bin are samples from one distribution, we calculate an empirical mean and variance of such distribution for bin *b* as:

(3) $$\mu_{b}=\frac{\sum_{i=(b-1)*s+1}^{b*s}{\hat{X}}_{i2}}{s},\;\sigma_{b}^{2}=\frac{\sum_{i=(b-1)*s+1}^{b*s}{\left({\hat{X}}_{i2}-\mu_{b}\right)}^{2}}{s}.$$

Note that in the rest of the text and all figures, we show standard deviation or stdev (*σ*) instead of variance ($\sigma^{2}$) as it is an essential statistic for the estimation of a variance-stabilizing transformation (section 2.2).

Given *B* mean and stdev estimations for *B* bins, we fit a spline to ${\left(\mu_{b},\sigma_{b}\right)}_{b\in \{1..B\}}$ pairs to estimate the mean-stdev relationship, $\sigma =\sqrt{h(\mu )}$.

Step 4: Given the estimated mean-stdev relationship *h* from Step 3, we estimate the variance-stabilizing transformation *t* based on Equation (2). Since *h* is not parametric, *t* is obtained by the numerical integral of *h*(*x*) over a set of *X* along a range of signals (asinh-spaced).

### 2.4 Supplementary materials and methods

Data sources, alternative methods, and evaluation metrics are provided in Supplementary Table S1, Supplementary Sections S1.2 and S1.4, respectively.

## 3 Results

### 3.1 VSS-Hi-C signals have a unit variance

First, we applied VSS-Hi-C on observed intra-chromosomal interactions of chromosome 12 in three experiments. As expected, we found that the variance of observations between two biological replicates varies among genomic bin pairs and depends on the magnitude of observations (Fig. 1b). Furthermore, this dependency is not similar in different experiments. This implies that each experiment requires a different variance-stabilizing transformation (Supplementary Fig. S1b) and thus a data-driven approach is required. Additional details on distance-, chromosome-, or experiment-specific VSS-Hi-C can be found in Supplementary Section S2.1.

We compared the variance instability (VI) of signals after different transformations for all three experiments. The lower value of this metric indicates a more stabilized variance of transformed signals. We found that VSS-Hi-C results in lower VI in all three experiments followed by another data-driven transformation, VST (Fig. 1d). These results indicate that a data-driven strategy is required for a variance-stabilization task and they outperform heuristic-based approaches with strict distributional assumptions like log, asinh and hft significantly. A VSS-Hi-C (log converge) is a linear transformation of VSS-Hi-C signals to converge to log function asymptotically and its VI does not change because this metric is invariant to the linear transformation.

To further explore the mean-variance relationship after transformations, we visualized the mean and standard deviation of VSS-Hi-C signals in Fig. 1c and other transformations in Supplementary Fig. S6. We observe that the standard deviation of VSS-Hi-C signals is around 1 for all signal intensities as opposed to VST signals (second best transformation based on VI metric) with lower standard deviation (The importance of variance’s absolute value is discussed in Supplementary Section S2.5). Furthermore, the VSS-Hi-C transformation is a monotonically increasing function, which preserves the ranking of signals—particularly the expected distance decay curve, where the ratio of interaction frequencies at each genomic distance decreases as the distance increases (Supplementary Fig. S7).

One advantage of signals with a unit variance is their visualization because the changes in signal intensities have the same meaning across the range of signal intensities. A typical first step of Hi-C analysis is a heatmap visualization of interaction frequencies. It is impractical to visualize a multi-scale organization of a chromosome with a linear color scale as it only distinguishes diagonal vs. off-diagonal interactions (Fig. 1f). Therefore, a log transformation is typically applied before visualization. However, as shown previously, log signals do not have constant variance which is ideal for linear color scale. We compared the visualization of log and VSS-Hi-C signals corresponding to intra-chromosomal interactions within a 12 Mb region. Both long- and short-range interactions are better distinguished in the VSS-Hi-C heatmap. However, long-range interactions in the log heatmap are blurry as the standard deviation of low signals is higher than expected (Supplementary Fig. S6a).

### 3.2 Supplementary results

We assessed the value of VSS-Hi-C signals in two Hi-C downstream tasks, calling subcompartments and TADs. We show the better or comparable performance of subcompartment callers given VSS-Hi-C signals (Fig. 1e, Supplementary Section S2.3), and that variance-stabilized signals preserve the performance of TAD callers (Supplementary Section S2.4).

## 4 Discussion and conclusion

Here, we presented the VSS-Hi-C pipeline, a transformation approach for data-driven variance stabilization of chromatin contact counts from Hi-C data. Our results show that VSS-Hi-C signals have a unit variance across a whole range of signal intensities that are more meaningful and interpretable and provide clearer visualization of multi-scale chromatin organization. Furthermore, a constant variance property improves the performance of clustering methods relying on Gaussian observed variables for identifying subcompartments. Also, it does not degrade the performance of most computational tools developed for raw Hi-C data.

VSS-Hi-C is an extension of VSS (Bayat and Libbrecht 2021) proposed for variance stabilization of ChIP-Seq assays to Hi-C assay. Our open-source R implementation works with a well-developed class (InteractionSet) and file format (.cool) for Hi-C data. In addition to Hi-C-specific interfaces, we included variance-stabilizing transformation functions taking two-column data frame (corresponding to two replicates of the same sample) as input to stabilize the variance of signals from other modalities.

The importance of variance stabilization has been proved for analyses including multiple samples like sample clustering (Love *et al.* 2014, Hafemeister and Satija 2019) and differential expression analysis (Law *et al.* 2014). To our knowledge, the value of variance-stabilized signals for analysis tasks within a sample is not explored widely. Here, we showed two Hi-C analysis tasks benefiting from signals with constant variance, visualization, and calling subcompartments. Exploring other tasks related to Hi-C data or other modalities that benefit from variance-stabilized signals is an interesting future work. For example, loop callers that rely on the enrichment of interaction frequencies compared to neighbor genomic bin pairs might benefit from VSS-Hi-C signals to call significant loops.

## Supplementary data

Supplementary data are available at *Bioinformatics* online.

Conflict of interest: None declared.

## Funding

This work was funded by NSERC (RGPIN/06150-2018), and Health Research BC (SCH-2021-1734), Compute Canada (kdd-445), and SFU Computing Science (N000265).

## Data availability

There are no new data associated with this article.

## Supplementary Material

btae715_Supplementary_Data
