# Supplementary material for: VSS-Hi-C: variance-stabilized signals for chromatin contacts
Source: Bioinformatics. 2024 Dec 10;40(12):btae715. doi: 10.1093/bioinformatics/btae715 (PMC11648998; doi:10.1093/bioinformatics/btae715)
Supplement: btae715_Supplementary_Data [file btae715_supplementary_data.pdf]

VSS-Hi-C: Variance-stabilized signals for chromatin contacts (supplementary information)

1 Supplementary materials and methods

1.1 Data sources

Table. S 1: Data sources.

Data type	Cell type	link
ChIP-Seq data	GM12878	CTCF: ENCFF710VEH, ENCFF473RXY, ENCFF833FTF, ENCFF002DAJ<br>RAD21: ENCFF002CPK, ENCFF753RGL<br>SMC3: ENCFF686FLD<br>HMs and DNase-Seq: 'E116-*.bigwig' files
	K562	CTCF: ENCFF002CEL<br>RAD21: ENCFF002CXU<br>SMC3: ENCFF041YQC
	IMR90	CTCF: ENCFF453XKM<br>RAD21: ENCFF195CYT
Hi-C data	GM12878	Biological replicate 1: HIC001-HIC018<br>Biological replicate 2: HIC019-HIC029
	K562	Biological replicate 1: HIC069-HIC070<br>Biological replicate 2: HIC071-HIC074
	IMR90	Biological replicate 1: HIC050-HIC054<br>Biological replicate 2: HIC055-HIC056
	H1Esc	Biological replicate 1: 4DNFILOL3RLR<br>Biological replicate 2: 4DNFIT5LC9RX
	HFF	Biological replicate 1: 4DNFI7HPMW1O<br>Biological replicate 2: 4DNFIAIBSEAG

1.2 Alternative variance-stabilizing transformations

Here we briefly describe alternative strategies for transforming Hi-C signals for the purpose of variance stabilization.

1.2.1 log and asinh

When the mean-variance relationship is $h(\mu) \propto \mu^2$, the variance-stabilizing transformation is proportional to the log function (Ahlmann-Eltze and Huber, 2023). This transformation is widely used to preprocess bulk and single-cell genomic data. We use a log transformation ($\log(x+1)$) as a heuristic alternative. Another heuristic transformation is a hyperbolic arcsine function $\operatorname{asinh}(x) = \ln(x + \sqrt{x^2+1})$ which is defined for all real values and is a popular substitute for shifted log transform.

1.2.2 ChromoR (Haar-Fisz transform)

ChromoR (Shavit *et al.*, 2014) is a computational tool for Hi-C data that employs Haar-Fisz transformation (explained in the following paragraph) and wavelet shrinkage methods to stabilize the variance and reduce the noise of observations, respectively. We use ChromoR (Haar-Fisz + denoising) and Haar-Fisz in our evaluations. The Haar-Fisz transform uses data from a single replicate, employing the assumption that neighboring genomic positions are pseudo-replicates of one another.

Haar-Fisz transformation Gaussianizes and stabilizes the variance of the inhomogeneous one-dimensional Poisson process Fryzlewicz and Nason (2004) using a modified version of forward and inverse Haar discrete wavelet transform (DWT).

The forward Haar DWT works as follows: given an input vector $v = (v_0, v_1, ..., v_{N-1})$ of size N, where N is a power of 2 ($N = 2^J$), they define $s^0 = v$ and recursively perform the following steps:

$$s^j = (s^j_0, s^j_1, ..., s^j_{2^{J-j}-1}),\ s^j_i = \frac{s^{j-1}_{2i} + s^{j-1}_{2i+1}}{2}, \tag{1}$$

$$d^j = (d^j_0, d^j_1, ..., d^j_{2^{J-j}-1}),\ d^j_i = \frac{s^{j-1}_{2i} - s^{j-1}_{2i+1}}{2} \tag{2}$$

for $j = 1, ..., J$. The elements of s^j and d^j represent the smooth and detail of the original vector v at scale 2^j. The detail vectors from all scales and the smooth element from the coarsest scale, $(s^J, d^J, d^{J-1}, ..., d^1)$, are enough to reconstruct the original vector v.

The inverse Haar DWT reverses the equations 1 and 2 to give:

$$s^{j-1}_{2i} = s^j_i + d^j_i, s^{j-1}_{2i+1} = s^j_i - d^j_i, \tag{3}$$

for $i = 0, ..., 2^{J-j} - 1$ where $j = J, ..., 1$. And, reconstructed smooth elements at the first layer are the original vector ($v = s_0$).

The Haar-Fisz transformation is very similar to the Haar DWT with the modification that at each scale j, it defines f^j after calculating s^j and d^j by

$$f^j_i = \begin{cases} 0 & \text{if } s^j_i = 0, \\ d^j_i / \sqrt{s^j_i} & \text{otherwise.} \end{cases} \tag{4}$$

And, inverse Haar DWT is applied on $(s^J, f^J, f^{J-1}, ..., f^1)$ rather than $(s^J, d^J, d^{J-1}, ..., d^1)$.

The reason that the Haar-Fisz transformation can stabilize the variance is based on the Fisz theorem Fisz (1955) that guarantees the variance of 1 for a specific function of two Poisson random variables with large and close expectations. The new reconstruction function of smooth elements using f elements is equivalent to that specific function. Therefore, if neighborhood elements in the smooth vectors are close to each other, the Haar-Fisz transformation guarantees a stabilized variance for the reconstructed vector.

We should sort the inhomogeneous chromatin contact counts to force the neighbor elements to be close to each other. Next, we should pad it with 0 to the length of a power of 2. We can apply the Haar-Fisz

transformation to the processed vector to stabilize its variance. We used 'hft' function from 'haarfisz' package in R to apply this transformation.

1.2.3 VST

An alternative data-driven variance-stabilizing transformation is a vst function from DESeq2 package (Love *et al.*, 2014) originally designed for RNA-seq gene expression data.

The DESeq2 method models within-group variability of gene i by the dispersion parameter α_i, which describes the variance of counts via $\text{Var}(K_{ij}) = \mu_{ij} + \alpha_i \mu_{ij}^2$, where K_{ij} and μ_{ij} are observed and expected read counts for gene i in sample j. Accurate estimation of α_i is not possible when number of samples is not large. Therefore, they employ empirical Bayes shrinkage for dispersion estimation.

Their procedure has 3 steps. First, they estimate gene-wise dispersions using maximum likelihood given observations for each gene. Then, they assume that there is a relationship between gene expression abundance and dispersion such that genes with similar average expression have similar dispersion. Therefore, they learn a trend between average gene expression and estimated dispersions from the first step. This provides an accurate estimate of the expected dispersion given the average expression of a gene. To account for both gene-wise dispersion estimations and expected one given a trend, they calculate final dispersions by shrinking gene-wise estimation towards the trend. The final dispersions together with mean expressions provide estimations of the variances ($\text{Var}(K_{ij}) = \mu_{ij} + \alpha_i \mu_{ij}^2$).

Then, variance-stabilizing transformation is obtained by numerical integration (equation 2 from main manuscript) of the spline fitted to the estimated mean and variance pairs.

We found that VST does not fully stabilize variance, perhaps due to producing separate estimates for each locus pair, requiring more replicates that are available for Hi-C data (Results).

1.2.4 VSS-Hi-C (log converge)

In order to place transformed signals in a familiar scale, the vst function from DESeq2 linearly scales the estimated transformation function to converge to the log function asymptotically. More formally, given a variance-stabilizing transformation function $t(x)$, they define $t'(x) = a \times t(x) + b$ such that $t'(x) = log(x)$ for large x values. Then, they use two x values, $x_1 = \text{quantile}(X, 0.95)$ and $x_2 = \text{quantile}(X, 0.99999)$ (X is a set of raw signal values), to define a system of equations $t'(x_i) = a \times t(x_i) + b = log(x_i)$ and find a and b. We employ a similar strategy and use VSS-Hi-C converging to the log function named VSS-Hi-C (log converge) in our evaluations. While the resulting units have a familiar scale and retain variance-stability, they lose the property of unit variance (Fig. S6f) and we found they perform slightly worse when input to downstream tools (Results).

1.3 Experiments details

All experiments, except for TAD analysis and performance assessments across resolutions, are conducted on Hi-C data at a 100 Kb resolution. The TAD analysis is performed at 50 Kb resolution.

The VSS-Hi-C hyperparameter, bin size, is set to 100 for all resolutions except for the 1 Mb resolution, where it is set to 20.

1.4 Evaluation metrics

1.4.1 Variance instability

To evaluate whether a given transformation method successfully stabilizes variance across different signal strengths, we employ the same binning strategy in VSS-Hi-C to group signals. We define the variance instability (VI) metric as the variance of variance of signals in each group. Therefore, more instability of variance results in higher VI.

More formally, given transformed signals, $t(\overrightarrow{x^{(\text{base})}})$ and $t(\overrightarrow{x^{(\text{aux})}})$, we use the same binning strategy introduced in section 2.3 to group $t(\overrightarrow{x^{(\text{aux})}})$ signals into B bins. Note that ordering by $t(\overrightarrow{x^{(\text{base})}})$ is the same as ordering by $\overrightarrow{x^{(\text{base})}}$ since t is a monotonic function. Assuming I_j be the set of positions in bin j, we define

$v_j = \mathrm{Var}_{i \in I_j}(t(\overrightarrow{x^{(\mathrm{aux})}})_i)$ as a variance of signals within bin j. Finally, we define the VI metric as the scaled variance of v_j across bins,

$$\mathrm{VI}(t) = \frac{1}{\sigma_1^2 \sigma_2^2} \mathrm{Var}(v_{1:B}).$$

The $\frac{1}{\sigma_1^2 \sigma_2^2}$ is a normalization factor to enforce $t(x)$ and $\alpha t(x)$ have the same VI value for a constant α. $VI(t)$ equals zero when the empirical variance is equal across all signal magnitudes; higher $VI(t)$ indicates worse residual variance instability.

1.4.2 Downstream analyses

Compartment analysis: The chromatin is segregated into two or more states ((sub)compartments), each enriched with specific properties like epigenomic, replication timing, and transcription patterns. Originally, two compartments, A and B, were identified based on their preferential interaction with each other (genomic bins within A compartment interact mostly with other genomic bins in A, and vice versa) (Lieberman-Aiden *et al.*, 2009). (Rao *et al.*, 2014) shows that two compartments can be further divided into different subcompartments with different epigenomic patterns by analysis of higher-resolution Hi-C data. They cluster genomic bins into subcompartments according to their long-range (inter-chromosomal) interaction profiles by the Hidden Markov model (HMM).

Following (Rao *et al.*, 2014), we use chromosomes 1 to 4 to create odd-even inter-chromosomal contact matrix, $C_{oe} = \begin{bmatrix} C_{12} & C_{14} \\ C_{32} & C_{34} \end{bmatrix}$, where C_{ij} is inter-chromosomal contact matrix between chromosomes i and j. Note that rows and columns of C_{oe} include genomic bins within odd and even chromosomes, respectively. Then, we apply a hidden Markov model (HMM), a clustering method with stable solution with Gaussian distributed variables, on C_{oe} and its transpose (C_{oe}^T) to annotate odd and even chromosomes respectively. (For comparison, we also tried a KMeans clustering method.) Finally, we biologically evaluate subcompartment annotations to assess the impact of transformations on finding more biologically meaningful subcompartments. To biologically evaluate the subcompartment annotation, we hypothesize that positions in the same subcompartment should have similar transcription factor binding and histone modification activity. Thus, following previous work (Libbrecht *et al.*, 2015; Shokraneh *et al.*, 2023), we calculate the variance explained metric (VE), a fraction of the variance of a relevant signal that is explainable by annotation. This metric is bounded by $[0, 1]$, and higher values indicate more agreement between annotation and signal values. We use 12 epigenomic signals including ChIP-seq targeting H2A.Z, 10 histone modifications and DNase-seq.

TAD analysis: Topologically associating domains (TADs) are diagonal blocks in Hi-C contact matrices representing self-interacting genomic regions. They have important functional roles like they are linked to gene regulation by exposing and insulating regulatory elements to gene promoters (Symmons *et al.*, 2016). Different algorithms exist to identify TADs from Hi-C contact matrices taking raw or normalized data as input (Zufferey *et al.*, 2018). Overall, TAD callers are based on linear scores, statistical models, network features or clustering. We use one TAD caller per category to assess the performance of these algorithms given transformed data: (1) TopDom (Shin *et al.*, 2016) is based on linear scores and had the best overall performance in a benchmarking study (Zufferey *et al.*, 2018), (2) SpectralTAD uses spectral clustering, it is efficient and its detected domain boundaries are enriched with biological features (Cresswell *et al.*, 2020), and (3) HiCseg (Lévy-Leduc *et al.*, 2014) is a statistical model which was also among the top scoring in a benchmarking study (Zufferey *et al.*, 2018).

We apply TAD callers on raw and transformed data to assess the validity of identified TADs after different transformations. Following a previous benchmarking study (Zufferey *et al.*, 2018), we evaluate TADs according to their enrichment of expected structural proteins, CTCF, RAD21, and SMC3, around TAD boundaries. This metric is a fold change between peaks around the boundary vs background regions. More formally, given a structural protein profile (SPP, including a number of peaks in 5 Kb intervals), we calculate 'peak' as the average SPP within a region surrounding boundary ($\pm$100 Kb) and 'background' as average SPP in two regions spanning 100 Kb each and located 400 Kb apart from the boundary (Zufferey *et al.*, 2018). Finally, we calculate fold change $\mathrm{FC} = \frac{\mathrm{peak}}{\mathrm{background}} - 1$ so FC greater than 0 indicates more enrichment of structural proteins around the boundary.

2 Supplementary results

2.1 Learning genomic distance-, chromosome- or genome-wide mean-variance and transformation curves

After demonstrating the experiment-specific mean-variance trend of intra-chromosomal interactions for chromosome 12 (Fig. 1a), we compared the mean-variance trend of intra-chromosomal interactions across different chromosomes within the same experiment for five separate experiments (Fig. S2a). We observed that chromosome-specific patterns were present, to varying degrees, particularly in three experiments from Rao et al. Rao *et al.* (2014): GM12878, IMR90, and K562. One possible reason for the differences between chromosomes could be the varying levels of similarity between them. When two replicates are less similar, ordering based on one replicate can lead to more scattered observations from the other replicate falling into the same bin. We calculated two metrics, hicrep Yang *et al.* (2017) and Pearson correlation, to indicate the similarity of biological replicates for each chromosome (Figs S2b, S2c). Our results confirm that the different trends observed among chromosomes are associated with the varying levels of similarity between replicates. For example, the chromosomes with the lowest standard deviation in the GM12878, IMR90, and K562 experiments are chromosomes 18, 22, and 22, respectively, which show higher similarities. Conversely, the chromosomes with the highest standard deviation in the GM12878, IMR90, and K562 experiments are chromosomes 19, 21, and 13, respectively, which exhibit the lowest similarities.

One hyperparameter of VSS-Hi-C is the auxiliary type, which indicates which replicate is being used as an auxiliary signal. Ideally, we expect the learned mean-variance relationships and transformation curves to be invariant to this hyperparameter. We estimated mean-variance pairs using each replicate as an auxiliary signal and compared the estimates across five Hi-C experiments (Fig. S3). Our results show similar trends among the estimates, except in experiments with the lowest sequencing depths and where there is a large disparity in sequencing depth between the two replicates (Table. S2). Generally, the replicate with lower sequencing depth has a smaller range of signals and, consequently, lower variances. Since all of our analyses are performed on one replicate, a common unit between replicates is unnecessary. However, if a common unit is required and estimates vary significantly between replicates, we recommend using the replicate with higher sequencing depth as the auxiliary signal, as it provides more accurate variance estimates, especially for larger signals that only appear in that replicate.

The genomic distance effect is a property of Hi-C data where the expected interaction between two genomic bins depends on the distance between them—the shorter the genomic distance, the higher the expected interaction frequency. Given that the mean is dependent on distance, it is unclear whether the variance is also directly influenced by distance. To test this hypothesis, we applied VSS-Hi-C to each genomic distance separately and estimated distance-specific means and variances (Fig. S5). Our results show that the mean-variance trend is consistent across genomic distances, confirming that VSS-Hi-C can be applied on a chromosome-wide scale without accounting for differences in genomic distance.

Some downstream Hi-C analyses, such as visualization and calling loops or TADs, are performed per chromosome pair, while others, like calling subcompartments, are conducted on genome-wide Hi-C data. Genome-wide analyses require shared units across all chromosome pairs, whereas chromosome pair analyses can use different units. We stabilize the variance of intra-chromosomal interactions on a per-chromosome basis, as all downstream analyses are performed at the chromosome level. This approach allows us to account for chromosome-specific mean-variance trends in intra-chromosomal interactions while requiring less memory and computational time. In contrast, we learn a genome-wide mean-variance trend for inter-chromosomal interactions, resulting in shared units across chromosome pairs, which is essential for calling subcompartments. Additionally, we demonstrated that mean-variance trends are not chromosome-pair-specific for inter-chromosomal interactions (Fig. S4).

2.2 VSS-Hi-C is scalable and stabilizes the variance across resolutions

The sequencing reads from Hi-C experiments are processed into Hi-C contact maps at different resolutions. Here, ”resolution” refers to the length of a genomic bin. The choice of resolution typically depends on the sequencing depth of the experiment and the downstream analysis. For instance, Hi-C contact maps at resolutions of 10 Kb, 100 Kb, and 1 Mb are used to identify loops or TADs, subcompartments, and compartments,

respectively. We assessed the performance and scalability of VSS-Hi-C across three resolutions.

VSS-Hi-C has a hyperparameter, bin size, which indicates the number of features or bin pairs that are grouped together to estimate the empirical mean and variance. Our results show that the performance of VSS-Hi-C remains consistent across a range of bin sizes from 20 to 100 at finer resolutions. However, for coarser resolutions, such as 1 Mb, a smaller bin size is required. This is due to the limited number of bin pairs at coarser resolutions. For example, on the smallest chromosome, there are fewer than 500 bin pairs at the 1 Mb resolution. Using a bin size of 100 would result in 5 estimated mean-variance pairs, which is insufficient to capture the underlying trend. As a result, we chose a bin size of 20 for the 1 Mb resolution and 100 for other resolutions.

Our findings indicate that VSS-Hi-C, followed by another data-driven approach, VST, outperforms other methods in stabilizing variance across all three resolutions (Fig. S8b). Additionally, we observed that the performance gap between data-driven and heuristic methods becomes more pronounced at finer resolutions. Heuristic transformations, such as log and asinh, tend to inflate variance for small counts, which is more common at finer resolutions due to the higher prevalence of small counts.

Next, we evaluated the scalability of VSS-Hi-C for finer resolutions. VSS-Hi-C can process chromosome 20 at a 10 Kb resolution in less than 30 seconds, making it faster than most methods, except for element-wise transformations like log and asinh (Fig. S9a). VSS-Hi-C is currently implemented in R, and the main bottleneck is the loading of .cool files into the InteractionSet object (see "Load" in Fig. S9a). A Python implementation of VSS-Hi-C could potentially enhance its scalability to finer resolutions, such as newer experiments like Micro-C (Fig. S9b).

2.3 VSS-Hi-C signals improve the performance of subcompartment callers

To evaluate the utility of transformed Hi-C data for annotating subcompartments, we apply two clustering methods, the Hidden Markov model (HMM) and KMeans on raw and transformed odd-even inter-chromosomal contact matrices (supplementary section 1.4.2). To evaluate called subcompartments, we hypothesize that positions in the same subcompartment should exhibit similar activity and measure their adherance to this hypothesis with the previously-described variance explained (VE) metric (Methods). Since researchers have reported different numbers of subcompartments, we show results for $K = \{3, 5, 7, 9\}$. We use Hi-C data for GM12878 cell line (Rao *et al.*, 2014) in this experiment because subcompartment annotation requires high-resolution Hi-C data. For data-driven transformations, VSS-Hi-C and VST, we use an inter-chromosomal contact matrix between chromosomes 1 and 2 to fit the mean-variance relationship and derive a transformation function. Then, we apply a transformation function to this and the rest of the matrices. For Haar-Fisz and ChromoR transformations, we transform data from all pairs of chromosomes together.

We observe that VSS-Hi-C (along with log and asinh transformations) outperform other transformations (Fig. S10). To better understand the impact of variance stability on this task, we calculated the variance instability (VI) metric for transformed inter-chromosomal data. We observe that VI for log and asinh signals is similar to VSS-Hi-C signals (Fig. S11a) as opposed to intra-chromosomal data (Fig. 1d). This might be due to the sparsity of inter-chromosomal data. Note that the robustness of estimated means, variances and their relationships in VSS-Hi-C depends on the reliability of the ordering based on another replicate. VSS-Hi-C can overestimate the variances when replicates are sparse and Aux read counts within a bin are less probable to be drawn from the same distribution. Therefore, VSS-Hi-C becomes slightly less advantageous in stabilizing the variance of inter-chromosomal data. However, it still performs comparably to log and asinh and outperforms other transformations.

Furthermore, we found that Haar-Fisz and ChromoR signals have the smallest variance instability while not improving the subcompartment annotations. This is probably because of the type of this transformation that is not a monotonic function as other transformations. Therefore, two genomic bin pairs with the same interaction frequency can have different transformed signal values (Fig. S11b), and the intensity of transformed signal values becomes less informative.

2.4 Variance stabilization preserves the performance of TAD callers

To evaluate the utility of transformed Hi-C data for identifying topologically associating domains (TADs), we apply three TAD callers, TopDom, HiCseg, and SpectralTAD on raw and transformed data with different transformations, and calculate the fold-change metric representing the enrichment of the expected structural proteins around TAD boundaries (supplementary section 1.4.2). We found that the enrichment of structural proteins around TAD boundaries identified by SpectralTAD (Fig. S12h) and TopDom (Fig. S12g) does not change after VSS-Hi-C transformation. Further comparison of a fold-change across all transformations (Fig. S12) shows that all transformations except Haar-Fisz preserve the performance of TAD callers.

Given a goal of variance-stabilized signals to fit the Gaussian assumption of statistical models, we expected the performance of HiCseg to increase after data transformation. However, we observed a small number of big TADs identified by HiCseg after variance-stabilizing transformations and they are not enriched with structural proteins (Fig. S13a). HiCseg is a dynamic programming (DP) algorithm that recursively calculates the likelihood of a segmentation. The analysis of a likelihood vs. the number of change points or boundaries shows that the optimum number of boundaries for variance-stabilized data is small (Fig. S13b). This is due to the assumption in HiCseg that all off-diagonal blocks (between-TAD interactions) have the same expected interaction frequency, μ_0. Consequently, with transformed data, the squared error corresponding to between-TAD interactions (difference between entries in off-diagonal blocks and a constant μ_0) is not negligible compared to the squared error corresponding to within-TAD interactions. Therefore, DP chooses larger TADs, less inter-TAD interactions, and less penalty from between-TAD interactions. Note that this is not a problem with raw data since the squared error for between-TAD interactions is negligible compared to the squared error for within-TAD interactions with large intensities and variances.

In general, we should pay attention to the assumptions of computational tools before inputting transformed data. Our experiments show that variance stabilization does not increase or decrease the performance of TAD callers without restrictive assumptions. However, a constant variance property can be uninteresting when the performance of a model depends on a lower variance for lower off-diagonal interaction frequencies.

2.5 The importance of variance instability and variance's absolute value of signals

The absolute value of variances across the whole range of signals is important in addition to VI (variance instability) which is a variance of variances because it implies the amount of information we keep from the original signal. To emphasize the importance of the absolute value of variances, consider a constant transformation function $t(x) = c$. This transformation has VI equal to 0 which is minimal while it ignores all the information from the original signal. Similarly, VSS-Hi-C (log converge) has a similar VI as VSS-Hi-C, however, the standard deviation of its signals is less than VSS-Hi-C (Fig. S6f). This can affect downstream analyses by putting less weight on signal differences.

2.6 VSS-Hi-C with one available replicate

VSS-Hi-C is applicable to datasets with two available biological replicates. In this section, we examine alternative approaches for data-driven variance-stabilizing transformations when only a single replicate is available and offer some suggestions. The original VSS paper Bayat and Libbrecht (2021) suggested two approaches: training a consensus transformation or using genomic autocorrelation. The first approach involves identifying experiment-specific parameters, such as the crosslinking method, digestion enzyme, and sequencing depth, which affect the mean-variance and transformation curves. A function could then be learned to map these parameters to the transformation function, allowing one replicate to be transformed based on its experimental parameters. However, it is unclear whether such a relationship exists or whether it can be learned from the available Hi-C experiments.

An alternative approach is to use autocorrelation in the genome. For example, neighboring regions on the linear genome are expected to have similar epigenomic states; therefore, they could be used as pseudo-replicates to apply VSS to 1-D epigenomic signals such as ChIP-Seq and estimate mean-variance pairs. In Hi-C experiments, we can consider two alternative binning or grouping strategies for VSS-Hi-C: grouping based on genomic distance or grouping spatial neighbors.

Each genomic distance has a specific expected interaction frequency in Hi-C contact matrices. Assuming that all observations within the same genomic distance are drawn from the same distribution, we can group all observations within that distance and calculate their means and variances. However, this is a strong assumption, as there are significant bin-pair-specific signals in addition to genomic-distance-specific signals, leading to overestimation of variances with this approach. For example, the estimated mean-variance pairs using this method (Fig. S14, left) show that the ranges of standard deviations are approximately three times greater than those of regular VSS-Hi-C (Fig. S1a), which groups bin pairs based on replicates to estimate mean-variance pairs.

We also investigated using spatial neighbors to group bin pairs. Specifically, we assumed that the interaction frequencies of bin pairs within an $n \times n$ Hi-C sub-matrix are drawn from the same distribution and used their means and variances to learn mean-variance and transformation curves. However, our results indicate that these estimates are not robust (Fig. S14, right). This lack of robustness is likely due to the presence of different types of 3D genome structures. For example, an $n \times n$ sub-matrix containing a loop structure would exhibit very high variance, as one bin pair may be expressed much more than its neighbors, while another sub-matrix could have very low variance if all bin pairs are expressed similarly.

Although we do not provide a method for learning a replicate-specific transformation, the simplest approach is to learn a single transformation curve from data with replicates and apply it across all datasets. This approach does not account for differences in the mean-variance relationship between datasets but, unlike alternatives such as log transformation, relies on a data-driven estimate.

3 Supplementary figures

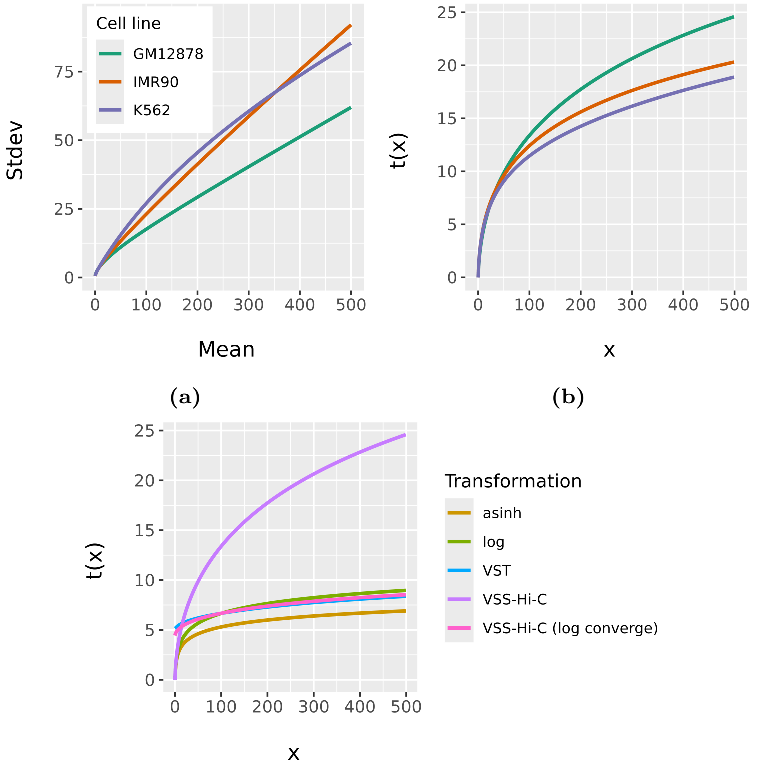

(c)

Fig. S1: (a) Learned mean-stdev curves for three Hi-C experiments, each from a different cell line. (b) Corresponding VSS-Hi-C transformations to mean-stdev curves in (a). (c) Comparison of different transformation methods.

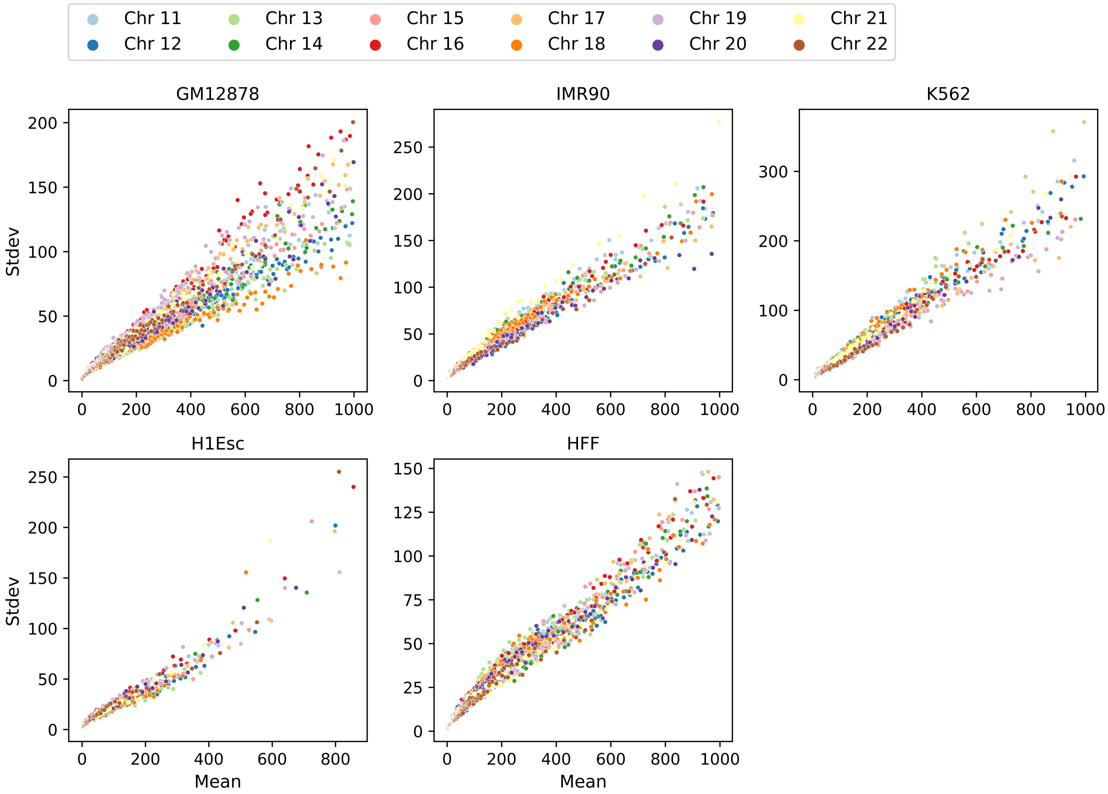

(a)

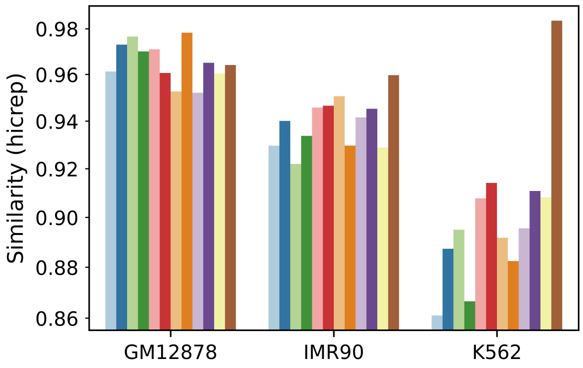

(b)

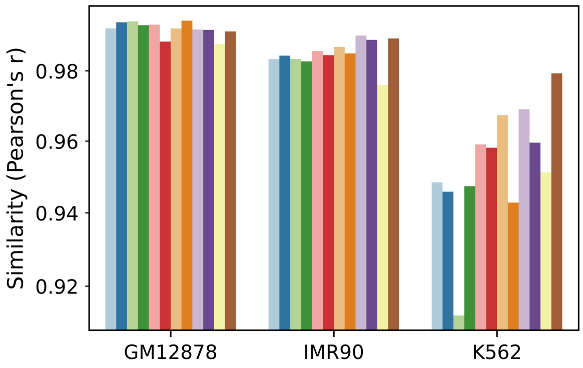

(c)

Fig. S2: (a) Comparison of mean-variance estimates inferred from intra-chromosomal interactions across different chromosomes for five experiments. (b, c) Similarity between two biological replicates of Hi-C contact maps for different chromosomes in three experiments, measured by (b) hicrep and (c) Pearson's r.

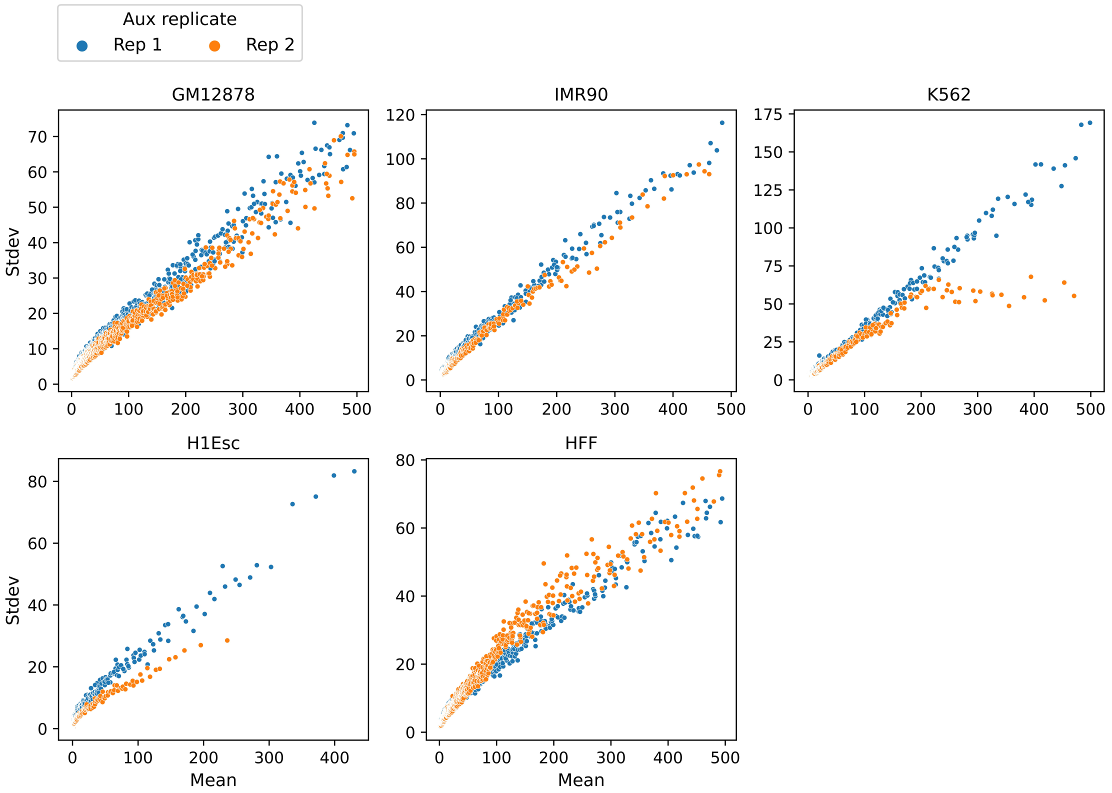

Fig. S3: Mean-variance estimates derived from two replicates of Hi-C data, using either Replicate 1 or Replicate 2 as an auxiliary replicate. This analysis is performed on chromosome 11 from the GM12878 cell line.

Table. S 2: Hi-C datasets statistics.

Cell line	library size (replicate 1)	library size (replicate 2)
GM12878	1.8 B	1.6 B
IMR90	460 M	271 M
K562	454 M	187 M
H1Esc	176 M	80 M
HFF	1.4 B	1.4 B

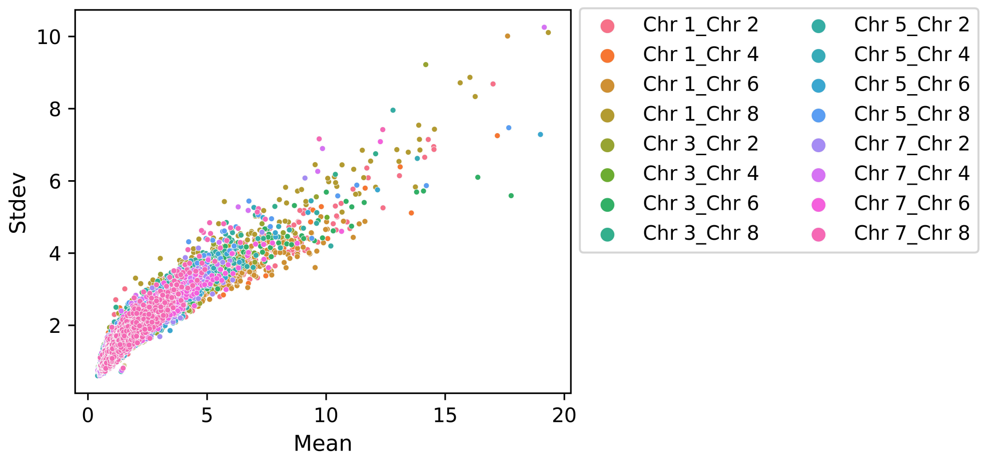

Fig. S4: Comparison of mean-variance estimates inferred from inter-chromosomal interactions of different chromosome pairs. This analysis was performed on the GM12878 cell line.

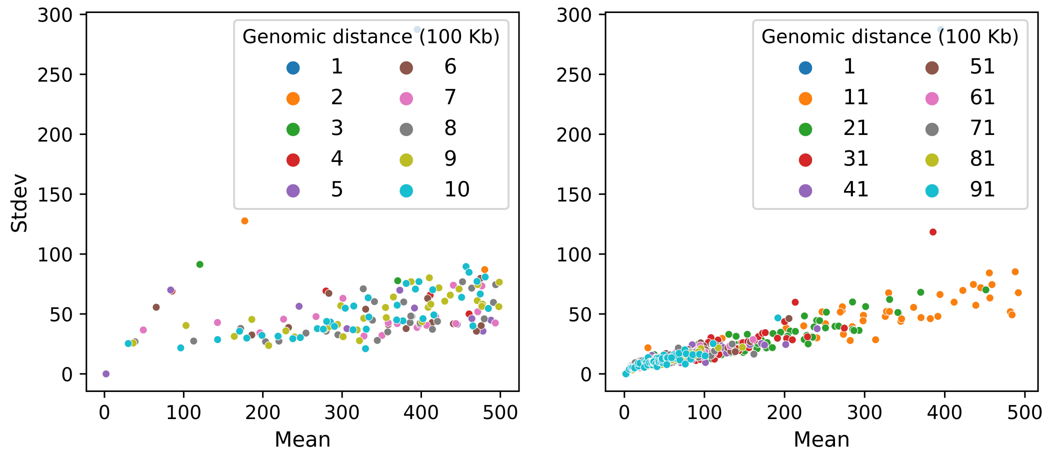

Fig. S5: Comparison of mean-variance estimates inferred from different genomic distances. Each plot corresponds to a different range of genomic distances. This analysis was performed on chromosome 11 from the GM12878 cell line.

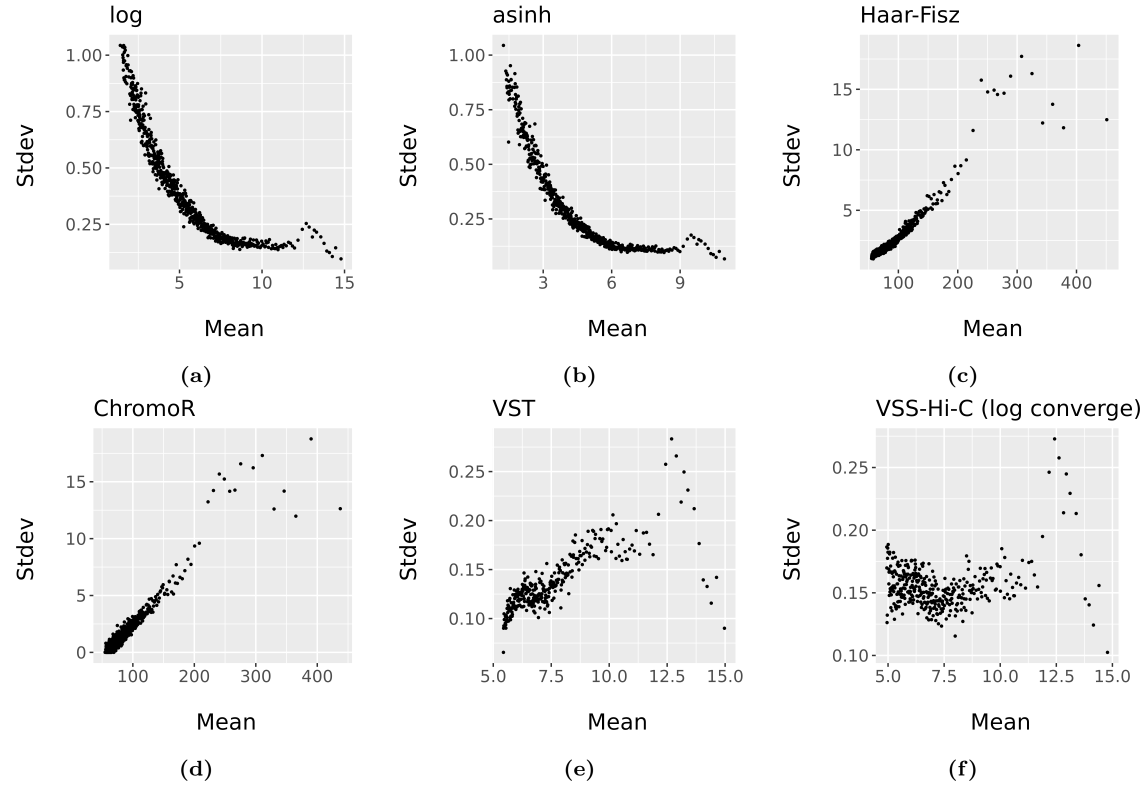

Fig. S6: Standard deviation (stdev) vs mean for transformed signals. These plots are for chromosome 13 of the GM12878 cell line.

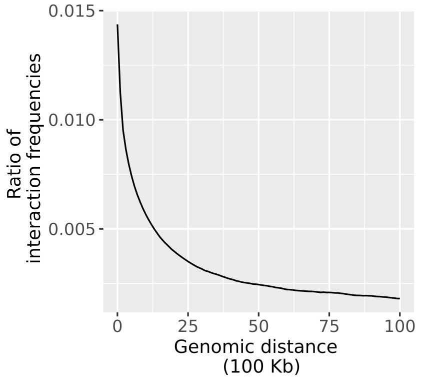

Fig. S7: Ratio of interaction frequencies at each genomic distance to the total interaction frequencies within the chromosome, after applying the VSS-Hi-C transformation. The analysis is conducted on chromosome 11 from the GM12878 cell line.

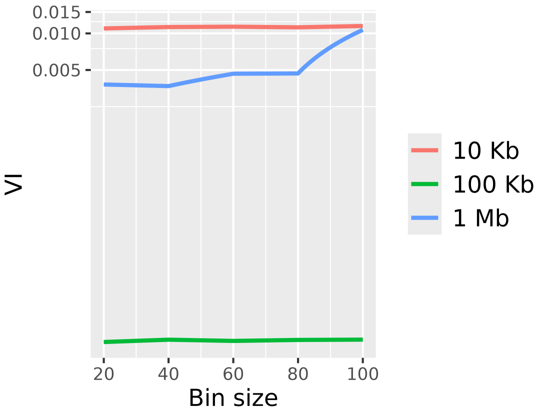

(a)

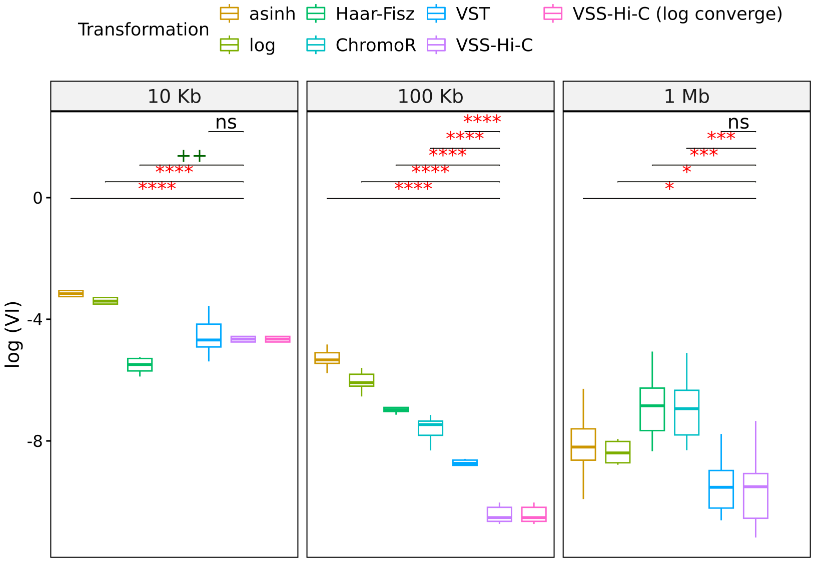

(b)

Fig. S8: (a) Analysis of the impact of VSS-Hi-C hyperparameter, bin size, on the performance of variance stabilization measured by Variance Instability (VI), where the lower value indicates the more stabilized variance. (b) Variance instability (VI) of transformed intra-chromosomal contact counts at three resolutions. Each point in box plots corresponds to one chromosome between chromosomes 17 to 22.

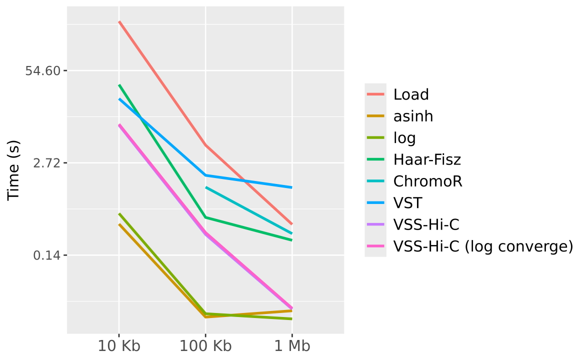

(a)

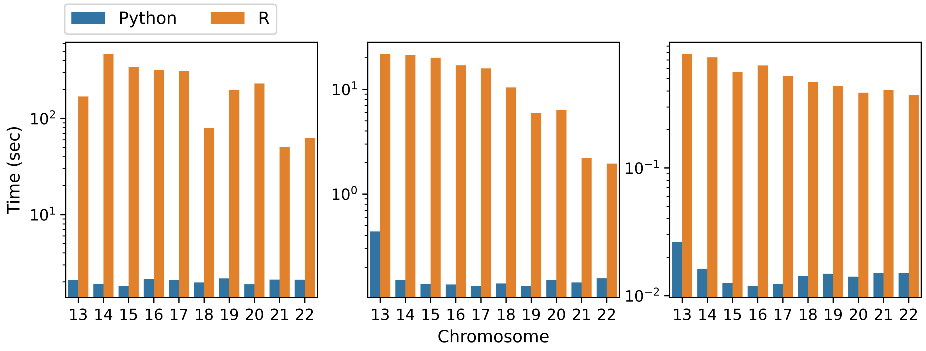

(b)

Fig. S9: (a) Comparison of the running time of transformation methods across three different resolutions, implemented in R. 'Load' refers to the time taken to load observations from the cool files. Running times correspond to the processing of chromosome 20 from the GM12878 cell line. (b) Comparison of load times between R and Python across chromosomes and resolutions.

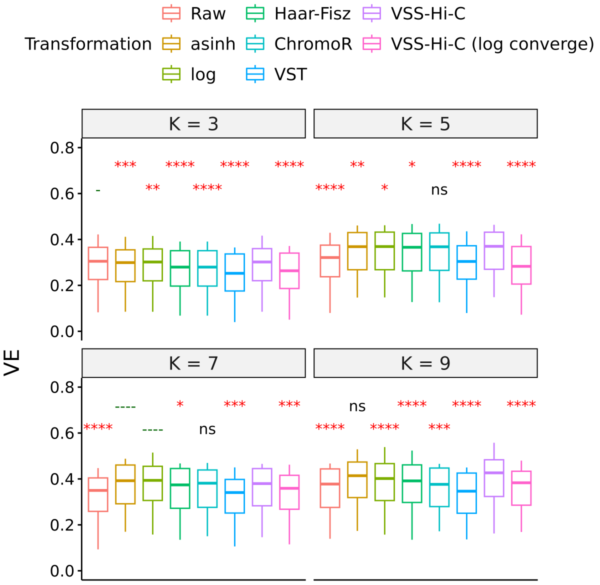

(a)

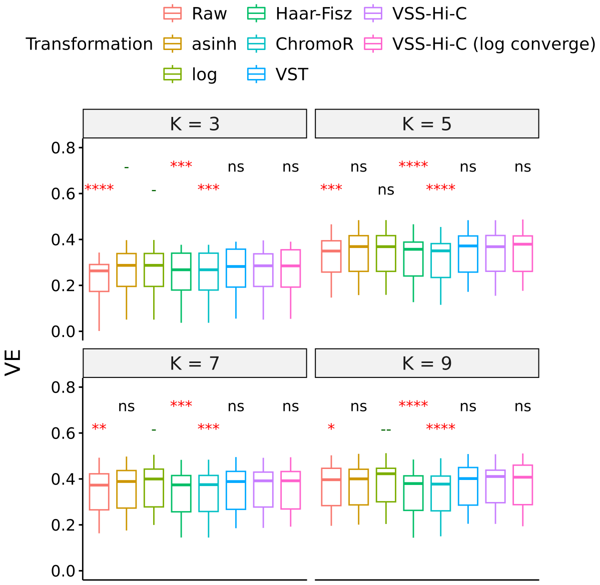

(b)

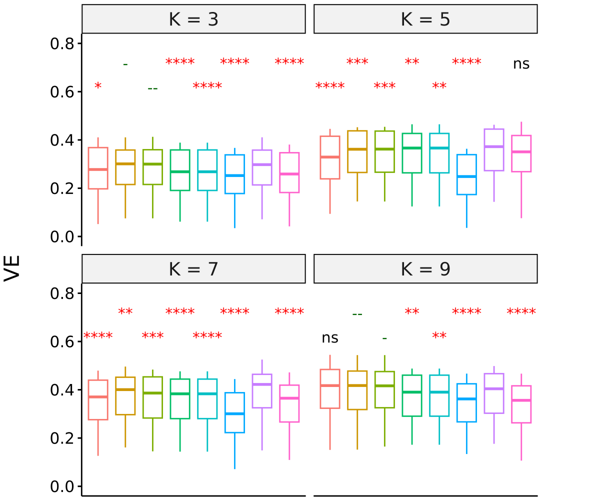

(c)

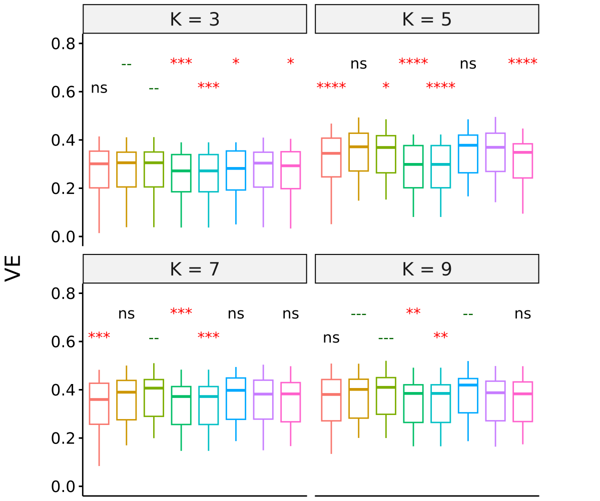

(d)

Fig. S10: Variance explained (VE) of epigenomic features given subcompartment annotations for (a) odd chromosomes by applying HMM, (b) even chromosomes by applying HMM, (c) odd chromosomes by applying KMeans, and (d) even chromosomes by applying KMeans, on raw and transformed odd-even inter-chromosomal matrix. K indicates the number of subcompartment types in the annotation. Each box represents 12 VEs corresponding to 12 epigenomic features for a specific transformation and K. This experiment is on the GM12878 cell line. Red asterisks indicate the significance of paired one-sided t-tests with an alternative hypothesis: VSS-Hi-C signals have higher VE compared to other transformed signals. Green dashes indicate the significance of tests with a reversed alternative hypothesis: VSS-Hi-C signals have lower VE compared to other transformed signals. Non-significant (ns) labels represent ($p > 0.05$) for both tests. *, **, ***, **** (-, --, ---, ----) represent p less than 0.05, 0.01, 0.001, and 0.0001 respectively.

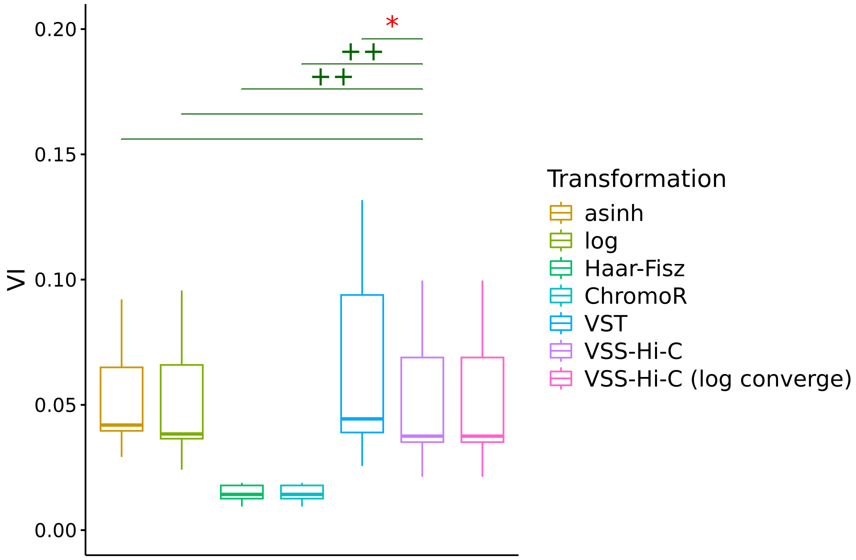

(a)

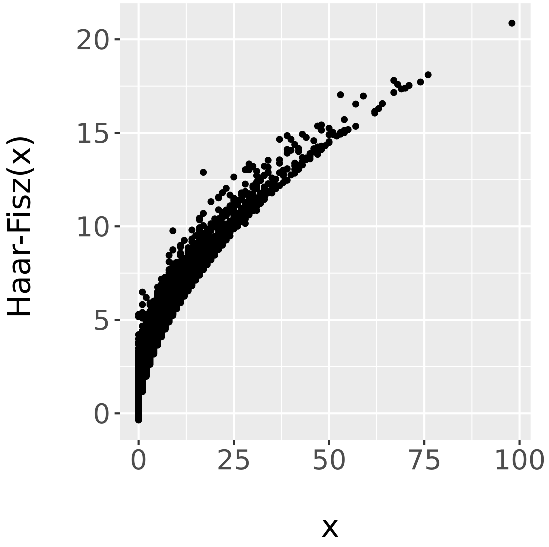

(b)

Fig. S11: (a) Variance instability (VI) of transformed inter-chromosomal Hi-C data. Red asterisks indicate the significance of paired one-sided t-tests with an alternative hypothesis: VSS-Hi-C signals have less VI compared to other transformed signals. Green pluses indicate the significance of paired one-sided t-tests with an alternative hypothesis: VSS-Hi-C signals have greater VI compared to other transformed signals. Non-significant labels ($p > 0.05$) are removed. *, **, ***, **** (+, ++, +++, ++++) represent p less than 0.05, 0.01, 0.001, and 0.0001 respectively. (b) Haar-Fisz vs raw signals for inter-chromosomal Hi-C data.

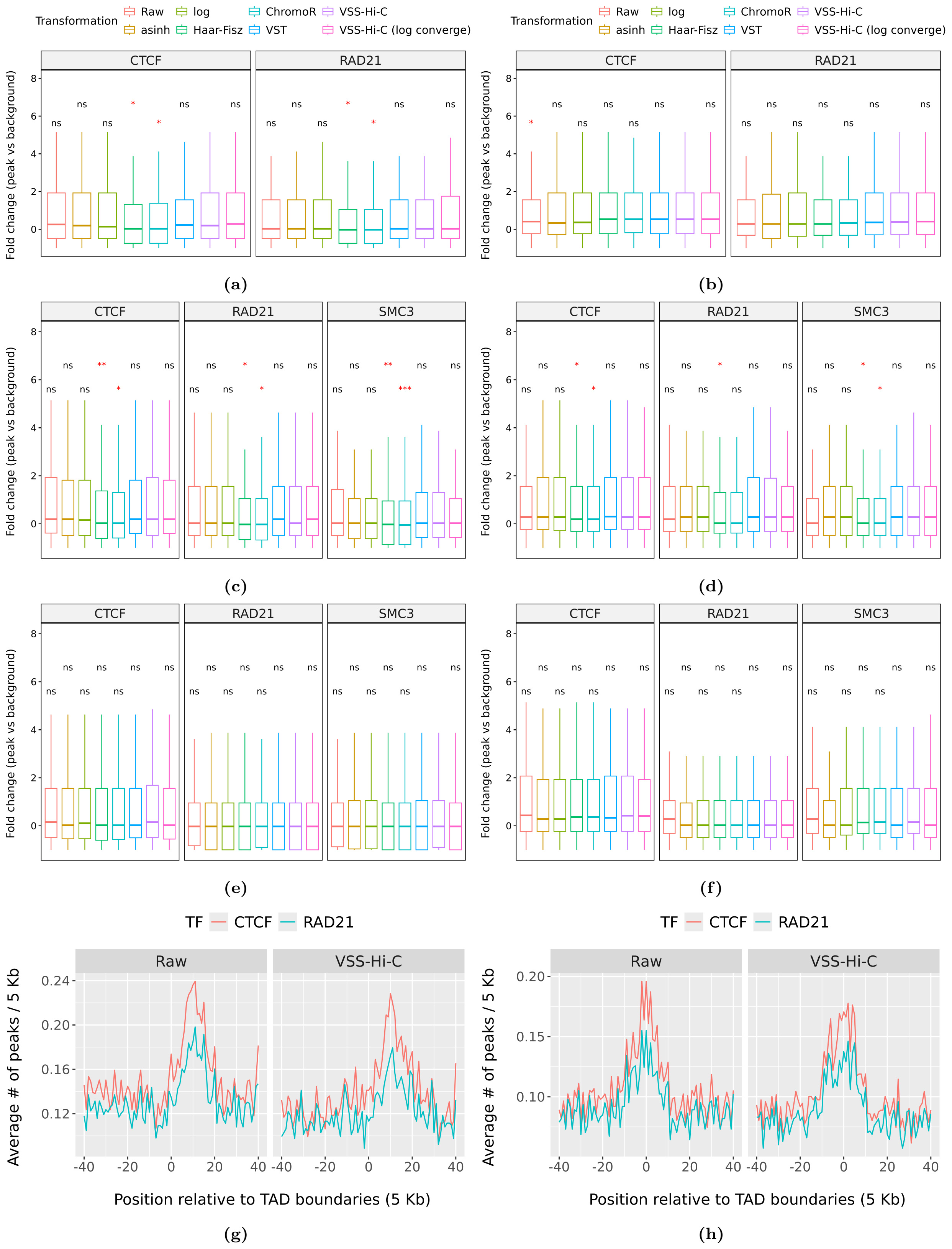

Fig. S12: (a-f) Fold change of CTCF, RAD21, and SMC3 peaks around TAD boundaries to distant regions for TADs identified by applying TopDom on (b) IMR90, (d) GM12878, (f) K562 and applying SpectralTAD on (a) IMR90, (c) GM12878 and (e) K562 cell lines. Red asterisks indicate the significance of one-sided t-tests with an alternative hypothesis: VSS-Hi-C signals have higher fold change compared to other transformed signals. *, **, ***, **** represent p less than 0.05, 0.01, 0.001, and 0.0001 respectively. 'ns' represents non-significant (p greater than 0.05). (g, h) Average number of ChIP-Seq peaks for CTCF and RAD21 transcription factors (TFs) per 5 Kb regions around (± 200 Kb) TAD boundaries identified by applying (g) TopDom and (h) SpectralTAD on raw and VSS-Hi-C signals (IMR90 cell line).

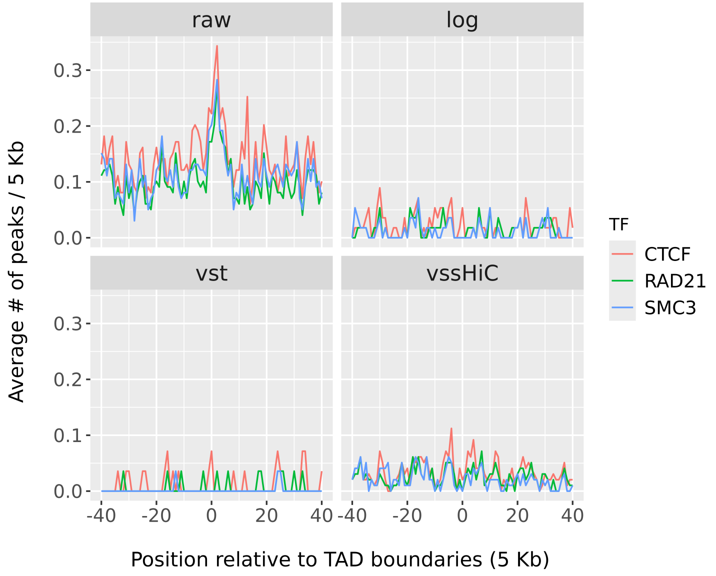

(a)

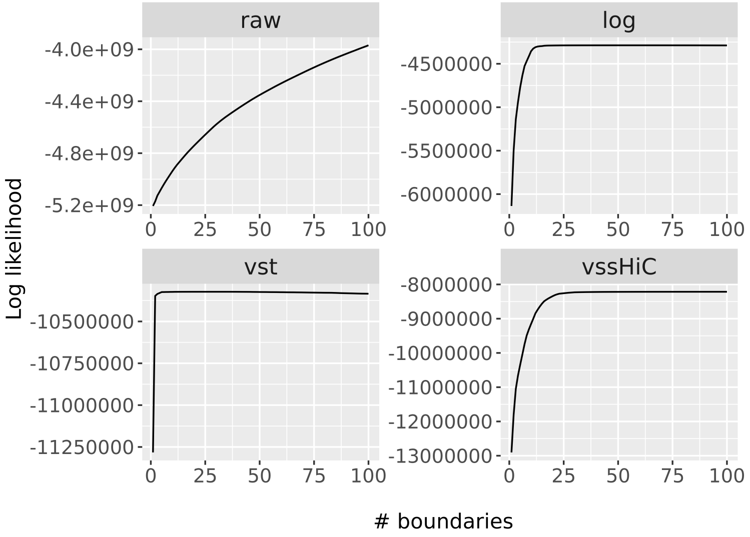

(b)

Fig. S13: (a) Average number of ChIP-Seq peaks for CTCF, RAD21, and SMC3 transcription factors (TFs) per 5 Kb regions around (± 200 Kb) TAD boundaries identified by applying HiCSeg on raw and transformed signals. (b) Log-likelihood of HiCSeg model vs number of change points or boundaries for raw and transformed signals. These plots are for GM12878 cell line.

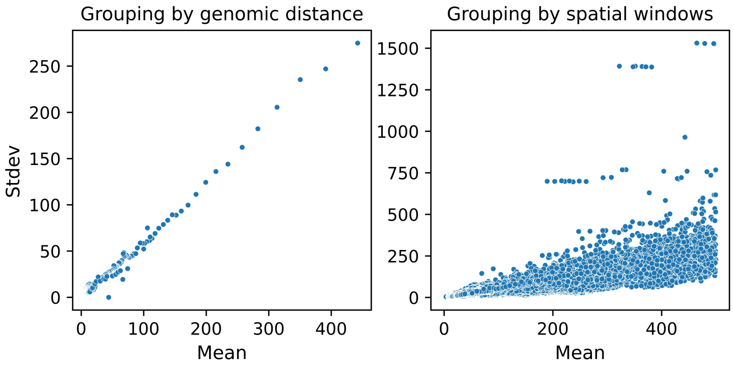

Fig. S14: Mean-variance estimates derived from one replicate of Hi-C data, driven by (a) grouping the bin pairs based on their genomic distance, or (b) using spatial neighbors in 3×3 sub-matrices as groups. This analysis is performed on chromosome 18 from the GM12878 cell line.

References

Ahlmann-Eltze, C. and Huber, W. (2023). Comparison of transformations for single-cell rna-seq data. *Nature Methods*, **20**(5), 665–672.

Bayat, F. and Libbrecht, M. (2021). Vss: variance-stabilized signals for sequencing-based genomic signals. *Bioinformatics*, **37**(23), 4383–4391.

Cresswell, K. G. *et al.* (2020). Spectraltad: an r package for defining a hierarchy of topologically associated domains using spectral clustering. *BMC bioinformatics*, **21**, 1–19.

Fisz, M. (1955). The limiting distribution of a function of two independent random variables and its statistical application. In *Colloquium Mathematicum*, volume 3, pages 138–146.

Fryzlewicz, P. and Nason, G. P. (2004). A haar-fisz algorithm for poisson intensity estimation. *Journal of computational and graphical statistics*, **13**(3), 621–638.

Lévy-Leduc, C. *et al.* (2014). Two-dimensional segmentation for analyzing hi-c data. *Bioinformatics*, **30**(17), i386–i392.

Libbrecht, M. W. *et al.* (2015). Joint annotation of chromatin state and chromatin conformation reveals relationships among domain types and identifies domains of cell-type-specific expression. *Genome research*, **25**(4), 544–557.

Lieberman-Aiden, E. *et al.* (2009). Comprehensive mapping of long-range interactions reveals folding principles of the human genome. *science*, **326**(5950), 289–293.

Love, M. I. *et al.* (2014). Moderated estimation of fold change and dispersion for rna-seq data with deseq2. *Genome biology*, **15**, 1–21.

Rao, S. S. *et al.* (2014). A 3d map of the human genome at kilobase resolution reveals principles of chromatin looping. *Cell*, **159**(7), 1665–1680.

Shavit, Y. *et al.* (2014). Combining a wavelet change point and the bayes factor for analysing chromosomal interaction data. *Molecular BioSystems*, **10**(6), 1576–1585.

Shin, H. *et al.* (2016). Topdom: an efficient and deterministic method for identifying topological domains in genomes. *Nucleic acids research*, **44**(7), e70–e70.

Shokraneh, N. *et al.* (2023). Integrative chromatin domain annotation through graph embedding of hi-c data. *Bioinformatics*, **39**(1), btac813.

Symmons, O. *et al.* (2016). The shh topological domain facilitates the action of remote enhancers by reducing the effects of genomic distances. *Developmental cell*, **39**(5), 529–543.

Yang, T. *et al.* (2017). Hicrep: assessing the reproducibility of hi-c data using a stratum-adjusted correlation coefficient. *Genome research*, **27**(11), 1939–1949.

Zufferey, M. *et al.* (2018). Comparison of computational methods for the identification of topologically associating domains. *Genome biology*, **19**, 1–18.
